# Hybrid AI Pipeline for Laboratory Detection of Internal Potato Defects Using 2D RGB Imaging

**DOI:** 10.3390/jimaging11120431

**Published:** 2025-12-03

**Authors:** Slim Hamdi, Kais Loukil, Adem Haj Boubaker, Hichem Snoussi, Mohamed Abid

**Affiliations:** 1 CES Laboratory, ENIS National Engineering School, University of Sfax, Sfax B.P. 3038, Tunisia; 2 LIST3N, Université de Technologie de Troyes, 10300 Troyes, France; 3 Eurocelp SAS, 14 Rue des Prés de Lyon, La Chapelle Saint Luc, 10600 Troyes, France; 4 EUT+ Data Science Institute, European Union; 5 Lab-STICC/ENSIBS, University of Bretagne du Sud, 56100 Lorient, France

**Keywords:** potato defect detection, deep learning, RGB imaging, YOLO, SAM, ResNet, random forest

## Abstract

The internal quality assessment of potato tubers is a crucial task in agro-laboratory processing. Traditional methods struggle to detect internal defects such as hollow heart, internal bruises, and insect galleries using only surface features. We present a novel, fully modular hybrid AI architecture designed for defect detection using RGB images of potato slices, suitable for integration in laboratory. Our pipeline combines high-recall multi-threshold YOLO detection, contextual patch validation using ResNet, precise segmentation via the Segment Anything Model (SAM), and skin-contact analysis using VGG16 with a Random Forest classifier. Experimental results on a labeled dataset of over 6000 annotated instances show a recall above 95% and precision near 97.2% for most defect classes. The approach offers both robustness and interpretability, outperforming previous methods that rely on costly hyperspectral or MRI techniques. This system is scalable, explainable, and compatible with existing 2D imaging hardware.

## 1. Introduction

The demand for automated, reliable, and scalable quality control systems in the agri-food industry has never been higher. As artificial intelligence (AI) reshapes laboratory inspection processes, the detection of internal defects in agricultural products presents a uniquely unsolved challenge. Among these, potato tubers stand out due to their anatomical variability and the economic impact of undetected internal anomalies during processing [1,2].

The global potato market is one of the most dynamic agricultural sectors, with more than 370 million tonnes produced annually, making the tuber the world’s leading non-cereal food crop by volume [3]. In Europe, potatoes play a central role in the agri-food industry, both for direct consumption and for laboratory transformation into high-value products such as chips, fries, and mashed potatoes.

Despite this strategic importance, quality control of tubers remains largely based on manual or semi-automated practices, particularly for internal quality assessment. In many European countries, the detection of internal defects such as hollow heart, bruising, or insect galleries still relies on visual inspections or destructive sampling methods. These techniques are poorly suited to current laboratory demands for speed, traceability, and standardization.

In response to these challenges, this work proposes a novel AI-based approach tailored for real-time, high-throughput, and low-cost defect detection using standard RGB 2D imaging, with the goal of bridging the gap between academic advances and laboratory applicability.

### 1.1. Overview of the Proposed Approach

To overcome the limitations identified in previous studies, we propose a novel hybrid and modular architecture specifically designed for the automatic detection of internal defects in potato tubers using standard RGB 2D slice imagery. Our approach combines the strengths of deep learning, classical machine learning, and expert-inspired logic to form a sequential decision-making pipeline that is both accurate and interpretable.

The proposed system begins with a high-recall object detection stage based on multiple YOLO models trained with class-specific confidence thresholds. Potential detections are then passed through a series of refinement modules, including patch-level classification using ResNet, semantic segmentation using the Segment Anything Model (SAM), and a contextual depth evaluation stage that leverages feature extraction via VGG16 and decision-making via a Random Forest classifier.

This layered architecture mimics human expert reasoning by progressively filtering, validating, and contextualizing each detection. It is designed to be robust to visual ambiguity and operational noise while remaining computationally efficient for real-time deployment in laboratory processing lines.

Unlike end-to-end black-box classifiers, our pipeline introduces transparency at each stage of inference, allowing for better understanding, easier maintenance, and targeted performance optimization. It also offers adaptability to evolving defect taxonomies and processing requirements, making it a viable and scalable solution for modern quality control systems in the agri-food sector.

**Novelty Statement.** The originality of the proposed system lies not in a single model, but in its **modular and interpretable design** that combines complementary deep and classical learning paradigms. Unlike traditional monolithic classifiers, our approach establishes a **multi-stage decision pipeline** integrating high-recall detection, patch-level verification, weakly supervised segmentation, and contextual depth reasoning. This configuration achieves both **laboratory scalability** and **scientific transparency**, bridging the gap between academic computer vision research and practical online quality control of agricultural products.

### 1.2. Problem Statement and Limitations of Existing Approaches

Detecting internal defects in potato tubers remains a major technological challenge, primarily due to the limited surface visibility of internal anomalies, the anatomical diversity of tubers, and significant variability across cultivars. In recent years, several approaches have been explored, including hyperspectral imaging [4,5], magnetic resonance imaging (MRI) [6], and RGB-based deep learning techniques [7,8]. While each of these methods offers promising results under controlled conditions, they exhibit several limitations that restrict their deployment in laboratory environments.

Hyperspectral imaging, although highly sensitive, relies on expensive, bulky equipment and requires precise calibration and lighting conditions, making it unsuitable for real-time, in-line applications [5]. Similarly, MRI-based techniques provide detailed structural insights but are restricted to laboratory use due to their high operational costs and complexity [6].

RGB-based computer vision methods represent a more cost-effective and scalable alternative. However, most existing solutions are based on monolithic classification architectures, often limited to binary outputs (defect/no defect), without any spatial localization or contextual reasoning. These models tend to be sensitive to noise, lighting variability, and artifact-prone cases. Moreover, they typically lack domain-specific logic or depth estimation capabilities, leading to high false-positive rates and poor interpretability in laboratory conditions [8].

Notably, none of the aforementioned approaches incorporate a multi-stage validation pipeline capable of refining ambiguous detections or distinguishing between morphologically similar defects, such as internal bruising versus insect galleries. These limitations underline the need for a modular, robust, and context-aware architecture specifically designed to meet the constraints of real-world potato processing lines.

### 1.3. Research Objectives

This study aims to develop a robust, modular, and laboratory-viable artificial intelligence (AI) architecture for the detection of internal defects in potato tubers using only standard RGB 2D imaging. The proposed solution is designed to address the key limitations of current approaches by combining high detection sensitivity, interpretability, and adaptability to varying conditions encountered in real production environments.

The main objectives of this work are as follows:

- To maximize the recall of internal defect detection while minimizing false positives, particularly for subtle or ambiguous anomalies.
- To enable precise localization and classification of multiple types of internal defects in a single pipeline (e.g., hollow heart, bruising, insect galleries).
- To ensure compatibility with real-time operation and laboratory constraints through the exclusive use of low-cost, high-speed RGB cameras.
- To design a multi-stage AI architecture incorporating detection, verification, segmentation, and contextual reasoning inspired by expert human inspection logic.

We aim to translate deep learning innovations into practical industrial tools by providing a scalable and explainable system for detecting internal potato defects during automated sorting.

### 1.4. Overview of the Proposed Architecture

To address the challenges outlined above, we propose a **multi-stage hybrid AI architecture** tailored to the detection of internal defects in potato tubers from standard RGB 2D slice images. The core of the system is designed to emulate the reasoning steps of human experts, while leveraging the complementary strengths of deep learning and classical machine learning.

The pipeline begins with a high-recall detection stage using multiple YOLO-based models trained with class-specific confidence thresholds. This ensures that even subtle or uncertain anomalies are flagged for further analysis. Regions with intermediate confidence scores are then re-evaluated using a ResNet-based patch classifier to improve precision and reduce false alarms.

To obtain fine-grained spatial information, the architecture incorporates the Segment Anything Model (SAM), which performs pixel-level segmentation within the YOLO bounding boxes. This weakly supervised coupling allows precise delineation of each defect region without additional manual annotation, a crucial advantage for laboratory datasets. The following stage performs a contextual evaluation to estimate the anatomical depth of each defect—specifically, whether it affects the outer skin or lies deeper in the parenchyma—using features extracted by VGG16 and classified via a Random Forest.

The final stage applies expert rule-based correction to merge, discard, or adjust detections according to laboratory inspection logic. Each module contributes a complementary function within the global decision process, resulting in a coherent, interpretable, and adaptive workflow.

Although the detection of internal defects in potatoes may appear visually simple, it is in fact a **highly challenging laboratory task**. Defects such as bruises, rust spots, or insect galleries exhibit **very low contrast, irregular textures, and strong visual similarity with healthy parenchyma**. Their appearance also varies significantly across **cultivars, lighting conditions, and slicing orientations**, which limits the generalization of conventional RGB-based models.

To overcome these challenges, our architecture establishes a **progressive reasoning chain** that integrates detection, verification, segmentation, and contextual interpretation:

- **Stage 1 (YOLO)**: high-recall detection with class-dependent thresholds;
- **Stage 2 (ResNet)**: patch-level reclassification to filter ambiguous cases;
- **Stage 3 (SAM)**: pixel-level segmentation within YOLO boxes for fine localization;
- **Stage 4 (VGG16 + Random Forest)**: contextual depth estimation (skin vs. flesh);
- **Stage 5 (Expert rules)**: final correction, merging, and validation.

This modular integration ensures a **step-by-step and interpretable reasoning process**, closely mimicking human visual inspection. Unlike previous single-stage approaches, the proposed system achieves **high precision, interpretability, and robustness to intra-class variability**, making it particularly suitable for real-world laboratory quality control.

### 1.5. Scientific Contribution and Positioning

The proposed approach introduces several key innovations that distinguish it from existing methods in the literature. Unlike traditional single-model solutions that rely solely on classification or basic segmentation, our pipeline integrates detection, reclassification, segmentation, and depth estimation into a coherent multi-level reasoning framework. This hybrid structure allows for both high sensitivity and interpretability, addressing practical laboratory needs such as robustness to noise, adaptability to defect variability, and compatibility with in-line operation.

Compared to hyperspectral imaging [4,5], magnetic resonance imaging [6], and conventional RGB-based CNN classifiers [7,8], our method offers:

- **laboratory scalability**, through the use of low-cost RGB cameras and real-time processing modules.
- **Defect-specific adaptability**, enabled by class-dependent YOLO thresholds and revalidation logic.
- **Context-aware analysis**, integrating both pixel-level segmentation and depth inference based on anatomical structure.
- **Interpretable decision-making**, by combining deep neural networks with classical classifiers in a modular pipeline.

To our knowledge, this is the first fully deployable architecture specifically designed for internal defect detection in potatoes that balances performance, interpretability, and ease of integration. As such, our work contributes both a methodological advance in agricultural computer vision and a practical solution aligned with the operational constraints of the food processing industry.

Our overarching goal is to bridge the gap between academic advances in deep learning and the practical requirements of the agri-food industry, providing a scalable and interpretable solution for internal quality assessment of potatoes on sorting and grading lines.

This work thus sets a new benchmark for RGB-based internal defect detection by combining precision, transparency, and full laboratory integration capabilities.

## 2. State of the Art

The automatic detection of internal defects in potato tubers has attracted growing attention over the past two decades due to its importance in reducing food waste, ensuring product quality, and meeting laboratory throughput requirements [4,9]. Several technological approaches have been explored to address this challenge, each offering trade-offs between accuracy, cost, speed, and interpretability. In particular, detecting internal anomalies such as hollow heart, internal bruising, or insect galleries remains difficult with conventional surface-based inspection.

Early methods relied on spectral imaging, including LED-based multispectral systems and hyperspectral imaging, which can reveal internal structures through specific wavelength interactions. More advanced techniques such as Magnetic Resonance Imaging (MRI) and X-ray tomography provide high-resolution internal scans but remain impractical for large-scale deployment due to cost and complexity. Recently, deep learning with RGB imaging has emerged as a promising alternative, offering faster and cheaper implementations, though often at the cost of interpretability and generalization [7,8].

In the following subsections, we critically review the main families of internal defect detection methods—multispectral imaging, hyperspectral imaging, MRI/X-ray-based systems, and RGB deep learning—highlighting their strengths, limitations, and suitability for laboratory use.

### 2.1. Multispectral and LED-Based Imaging

Some studies have explored the use of narrow-band LED illumination combined with 2D cameras to reveal contrast between healthy and defective tuber tissues. These systems exploit specific absorption or scattering properties of tuber flesh at wavelengths such as near-infrared or blue light [10]. For example, Zheng et al. (2023) demonstrated a line-scan multispectral system with deep learning that improved defect detection accuracy significantly [11]. Zhang et al. (2019) used a single-shot multispectral camera spanning 676–952 nm and achieved  91% classification accuracy across defect types such as scab, greening, and bruises [12]. Similarly, Deng et al. (2023) combined high-definition multispectral imaging with deep neural networks to detect multiple food defects, including on potatoes, with high precision [13]. Moreover, Semyalo et al. (2024) applied visible–SWIR spectral analysis (400–1100 nm) and obtained  91% accuracy in distinguishing internal defects like pythium and internal browning, helping to assess internal defect areas quantitatively [5].

Although these LED-based multispectral systems offer advantages in cost, compactness, and acquisition speed, they commonly suffer from several limitations:

- **Spectral ambiguity**: restricted bands reduce the ability to differentiate deeper or subtle internal defects.
- **Environment sensitivity**: performance drops under variable lighting, moisture, or depending on tuber variety conditions.
- **Calibration dependence**: each new dataset or operating context typically requires recalibration [14].
- **Limited generalization**: models trained on one cultivar or harvest season often fail to generalize to others [12].

Overall, while LED-based multispectral imaging offers a strong foundation for inline, non-destructive quality control, its current robustness and generalizability remain insufficient for fully scalable laboratory implementation without enhancements like adaptive band selection, intelligent calibration, or hybrid processing pipelines.

### 2.2. Magnetic Resonance Imaging (MRI) and X-Ray Techniques

Magnetic Resonance Imaging (MRI) and X-ray imaging have been widely used in laboratory research to visualize the internal structure of potato tubers with high spatial accuracy [15]. These modalities provide detailed insights into tissue composition, growth patterns, and defect morphology. For instance, ref. [16] used a 1.5 T MRI scanner to monitor the progression of internal rust spots over 33 weeks after harvest, using spatialized multi-exponential T_2_ relaxometry to non-destructively track tissue degradation. Similarly, ref. [17] demonstrated how spatially resolved T_2_ relaxation mapping can distinguish up to six tissue classes within stored tubers, including cortex, pith, and defect regions.

Beyond MRI, X-ray computed tomography (CT) has been employed to monitor diel growth and internal structural variation in response to environmental conditions [18]. In earlier studies, ref. [19] enhanced hollow heart detection by submerging tubers in water during radiography, improving contrast between healthy and defective zones. A comparative study by [20] evaluated MRI and X-ray alongside optical spectroscopy, concluding that while MRI offers superior accuracy, its throughput and cost hinder laboratory deployment.

Despite their precision and research value, these imaging techniques face major limitations for real-time laboratory use:

- **High cost and bulk**: MRI and CT systems are expensive, bulky, and require dedicated infrastructure.
- **Low throughput**: A typical MRI scan processes only 12–18 tubers in about 30 min [16].
- **Safety and regulatory constraints**: X-ray systems require shielding, operator certification, and legal compliance.
- **Operational complexity**: MRI and CT data require expert interpretation and advanced image processing pipelines.

In summary, although MRI and X-ray offer unmatched imaging resolution for internal defect detection, their practical application in high-throughput laboratory sorting lines remains limited due to technical and economic constraints.

### 2.3. Deep Learning with RGB Imagery

More recently, RGB-based deep learning methods have emerged as a promising compromise between cost and performance, leveraging standard RGB cameras and convolutional neural networks (CNNs) to detect internal defects with high speed and affordability. Early work by Hassan and Al. [7] and Moallem et al. [8] used CNN classifiers on 2D cross-sectional images or external cues to infer internal anomalies. However, these single-shot classifiers often lack spatial reasoning and interpretability.

The adoption of advanced detection and segmentation frameworks has since grown:

- **R-CNN/Fast R-CNN**: These architectures have been used for tuber segmentation and defect localization, but tend to be slow and resource-intensive in inference.
- **Faster R-CNN**: ResNet-based models have achieved ∼98% accuracy in surface defect detection via transfer learning (e.g., SSD Inception V2, Faster R-CNN ResNet101) [21].
- **Mask R-CNN**: Applied to segment potato tubers in soil, with detection precision around 90% and F1 ≈ 92
- **SSD (Single Shot MultiBox Detector)**: Fine-tuned for potato surface defects, achieving ∼95% mAP [21].
- **YOLOv5 and variants**: Including DCS-YOLOv5s, tailored for multi-target recognition in seed tubers (buds, defects), delivering fast real-time detection (∼97% precision) [22].
- **YOLOv10/11**: Emerging models; HCRP-YOLO achieved ∼90% true positive rates for germination defects.
- **Survey on YOLO evolution**: Recent reviews highlight improvements in speed and accuracy from YOLOv1 to YOLOv10, especially in agricultural scenarios [23].
- **Lightweight YOLOv5s variants**: Designed for laboratory defect detection with real-time performance on production lines [24].

Despite substantial advances, RGB-based pipelines still suffer from:

- **Black-box behavior**: Limited interpretability and anatomical reasoning.
- **Single-shot limitations**: Most approaches perform classification/detection in one pass without refinement.
- **Lack of context**: Models often only see external surfaces or slices, missing 3D anatomical structures.
- **Generalization gaps**: Performance typically drops when applied to new cultivars, lighting, or environments.

Nevertheless, the integration of two-stage or multi-stage architectures (detection → segmentation → refinement), interpretability modules, and anatomical priors presents a promising path forward—motivating the development of more modular, explainable, and robust RGB pipelines.

### 2.4. Unsupervised Anomaly Detection

In recent years, several unsupervised and self-supervised anomaly detection frameworks have been proposed to reduce dependence on extensive labelled datasets. Notable approaches include the Normal Feature-Enhanced Reverse Teacher–Student Distillation (NFERD) method [25] and asymmetric reverse-distillation techniques for industrial inspection [26]. While these approaches have shown impressive performance in binary abnormality localization tasks, they are typically designed for flagging anomalous regions rather than recognising the specific type of defect. In contrast, our supervised hybrid architecture is tailored to distinguish between distinct potato defect categories (e.g., hollow heart, insect galleries, and greening), thereby providing semantic classification rather than mere anomaly detection. Nevertheless, these unsupervised paradigms remain highly relevant, and future extensions could integrate them to enhance generalisation under limited labelling conditions.

### 2.5. Limitations and Motivation for a New Approach

Despite substantial progress in the detection of internal potato defects, no existing method fully meets the combination of laboratory constraints such as **low hardware cost**, **high throughput**, **anatomical interpretability**, and **robustness under real-world conditions**.

**Hyperspectral imaging**, while powerful in laboratory settings, is constrained by its high equipment cost, slow data acquisition, and the need for expert calibration. **MRI and X-ray techniques** offer precise visualization of internal tissue but are unsuitable for real-time laboratory processing due to their bulk, safety concerns, and high cost. **Multispectral systems** using LEDs are lightweight and affordable but lack sufficient spectral richness for consistent detection across cultivars and environments.

**Deep learning approaches based on RGB images** have brought promising advances, especially in terms of speed and flexibility. However, most existing RGB-based models are **single-shot classifiers or detectors** that:

- Offer **limited interpretability**, often functioning as black boxes.
- Are sensitive to **visual noise** and lack contextual anatomical reasoning.
- Do not perform **multi-stage refinement** to correct or validate uncertain detections.

To address these gaps, our work proposes a modular, interpretable, and scalable architecture that **unifies detection, verification, segmentation, and contextual anatomical analysis** within a single RGB-only pipeline. Each stage contributes complementary reasoning: from high-recall defect detection to patch-level verification, precise spatial segmentation, and depth-aware classification, mimicking human inspection logic.

Recent research in anomaly and defect detection has increasingly focused on **unsupervised, explainable, and hybrid learning frameworks**, highlighting the shift toward modular and interpretable architectures. Ref. [27] proposed unsupervised model for anomaly detection with compactness loss as classifier for drone and distance mahalanobis for fix cameras. Ref. [28] proposed a **multi-directional feature aggregation network** for unsupervised surface anomaly detection, improving localization under limited data conditions. Ref. [29] developed a **dual-branch attention mechanism** for interpretable fruit disease segmentation, demonstrating the benefits of multi-scale fusion in agricultural vision tasks. Ref. [30] presented **transformer–CNN hybrid models** combining global attention and local convolutional reasoning to achieve robust and explainable agricultural image analysis. These recent advances further support our motivation to design a **hybrid and interpretable architecture**, bridging deep learning and classical reasoning for reliable laboratory defect detection.

This comparison in Table 1 highlights the novelty of our contribution, which seeks to combine the affordability and speed of RGB imaging with the multistage intelligence typically reserved for more complex and costly systems. Our pipeline is therefore well-positioned for laboratory deployment in real-time sorting and quality control scenarios.

## 3. Proposed Method

This section presents the proposed hybrid pipeline for the detection of internal defects in potato tubers using RGB 2D imaging. The pipeline has been specifically designed to meet laboratory requirements: high throughput, low hardware cost, robustness to real-world conditions, and interpretability. Our method combines high-recall detection with multi-stage refinement, mimicking human expert reasoning. The pipeline consists of five stages: (1) YOLO-based detection, (2) patch reclassification, (3) semantic segmentation, (4) depth estimation, and (5) expert rule-based correction.

To formalize the processing workflow, Algorithm 1 describes the main steps of the proposed hybrid AI pipeline. It details how YOLO detection, ResNet-based patch validation, SAM segmentation, and contextual Random Forest reasoning are integrated to form an explainable decision chain.
**Algorithm 1:** Hybrid AI Pipeline for Internal Potato Defect Detection
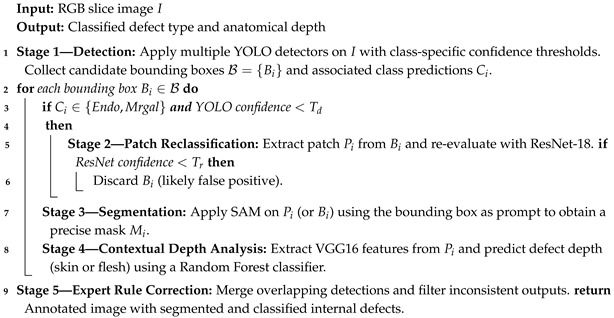


### 3.1. Dataset Description

**Figure 1:**
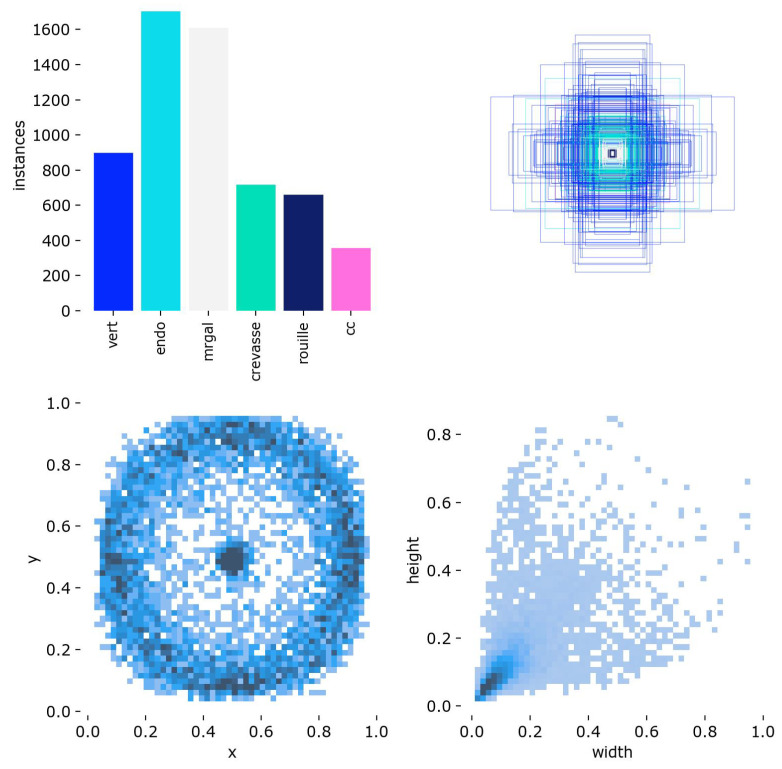
Distribution of YOLO annotations in the dataset. The majority of annotated defects are ‘Endo’ and ‘Mrgal’. Most bounding boxes are small (w,h<0.3), and centered around the core of the tuber.

The dataset consists of 2D RGB images of potato slices captured in a real laboratory environment. Each image was manually annotated in the YOLO format, with bounding boxes and class labels representing internal defects (Figure 1).

A total of over 6000 bounding boxes were labeled across six main classes:

- **Hollow Heart (cc)**—central voids with regular contours.
- **Damaged Tissue (Endo)**—internal bruising or blackened zones.
- **Insect Galleries (Mrgal)**—small tunnels or pest bites.
- **Cracks (Crevasse)**—structural fractures through the tuber flesh.
- **Rust Spots (Rouille)**—oxidized tissue lesions, typically subcutaneous.
- **Greening (Vert)**—green zones near the skin due to light exposure.

All annotations use the YOLO format <class_id> <x_center> <y_center> <width> <height>, with normalized coordinates. Most defects are small and centered, requiring high sensitivity from the detection module.

**Figure 2:**
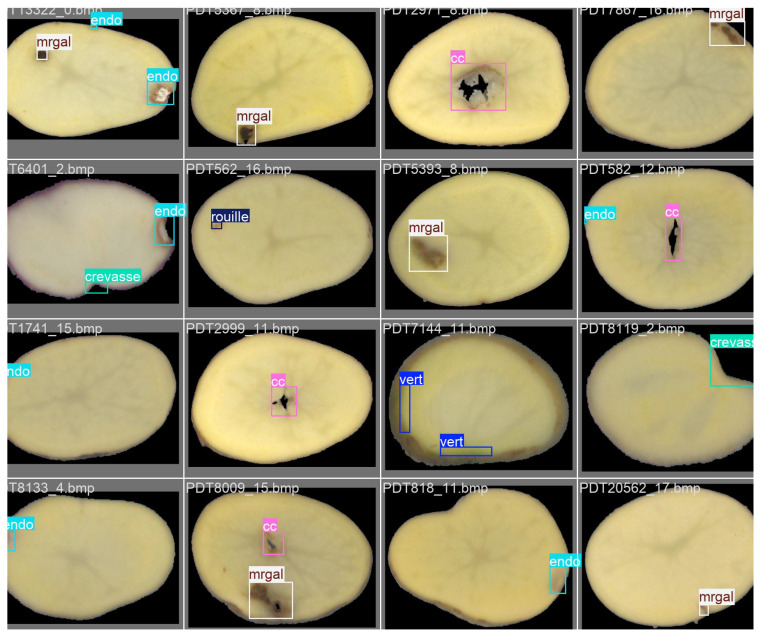
Representative potato slice images annotated with bounding boxes indicating the position and class of the detected defect. The considered classes include: *mrgal* (internal gallery), *endo* (internal damage), *cc* (hollow heart), *crevasse*, *rouille* (rust spot), and *vert* (green tissue). The figure highlights the wide variability in defect size, shape, contrast, and location, which underscores the challenges of building a robust detection pipeline suitable for laboratory deployment.

This analysis supports the need for a sensitive and localized detector, and motivates the design of a class-specific detection pipeline described in the following sections. Representative examples of annotated potato slice images are shown in Figure 2. These samples illustrate the diversity of internal defects considered in this study, as well as the variability in their visual appearance across the dataset.

This dataset is expected to become a reference resource for potato quality control research. It encompasses a wide range of samples collected from multiple potato varieties, including both yellow- and red-fleshed cultivars, thereby capturing the natural diversity encountered in laboratory practice. Its richness and variability provide a solid foundation for benchmarking and developing advanced AI-based approaches for defect detection and grading.

Table 2 summarizes the datasets employed in recent potato defect detection studies. While most existing works are trained and evaluated on small, homogeneous datasets (2–4 defect categories and texture variability), the proposed **Hybrid AI Pipeline** is assessed on a more challenging dataset of over **6600 laboratory RGB images**.

In this Table 2 the presence of **six heterogeneous defect types**—*Hollow heart, Internal damage, Insect galleries, Cracks, Rust spots, and Greening* table—significantly increases intra-class variability and inter-class confusion.

This makes our dataset one of the most comprehensive and complex benchmarks currently available for internal potato defect detection, providing a more realistic assessment of model robustness and generalization ability.

### 3.2. Architecture

The proposed hybrid pipeline is designed to mimic the reasoning process of human experts in defect inspection. Instead of relying on a single monolithic classifier, our system follows a modular sequence of specialized components. In this context, the term patch refers to the bounding boxes generated by the YOLO detector, which are dynamically adapted to each defect’s geometry rather than fixed-size image tiles. The size of these regions may vary considerably across defect types, from small insect galleries to large bruised areas. In practice, partially truncated defects are rare in potato slices, and no specific merging procedure was required in the current implementation. Each stage contributes complementary information, progressively refining the detection, verification, and contextual interpretation of potato defects. This modularity ensures robustness, interpretability, and adaptability for laboratory deployment.

#### 3.2.1. Stage 1: Initial Detection with Multi-YOLO

The pipeline begins with a high-recall detection stage based on multiple YOLO models trained in parallel. Each model is fine-tuned with class-specific confidence thresholds in order to maximize recall and ensure that even subtle or ambiguous anomalies are flagged. This stage outputs bounding box candidates corresponding to potential internal defects such as hollow heart, bruising, or insect galleries.

The training of the YOLO models was performed over 300 epochs with an early stopping patience of 60 epochs to prevent overfitting. A batch size of 8 and an image resolution of 640× 640 pixels were used, leveraging GPU acceleration on a CUDA-enabled device. We employed the Adam optimizer with an initial learning rate of $9.5\times 10^{-4}$, a cosine learning rate schedule with a final learning rate factor of 0.0103, and weight decay of $6.1\times 10^{-4}$. Momentum was set to 0.868 with a warm-up phase of 2.3 epochs and a warm-up momentum of 0.95. A dropout rate of 0.3 was applied to improve generalization.

Data augmentation played a key role in improving robustness. We applied hue, saturation, and value shifts (h=0.10, s=0.10, v=0.10), random translations (±8.7%), scaling (up to 55%), and horizontal flipping with a probability of 0.51. Mosaic augmentation was strongly emphasized (probability 0.97), while mixup and copy-paste augmentations were disabled. These strategies increased the diversity of training samples and allowed the models to handle the high intra-class variability observed in potato defects.

Loss function weights were tuned with values of 3.98 for the bounding box regression term, 0.54 for the classification term, and 1.20 for the distribution focal loss. The IoU threshold for positive matching during training was set to 0.4. Training was initialized from pretrained weights to accelerate convergence, with deterministic mode enabled for reproducibility.

This combination of hyperparameters, together with multi-model training, provided a strong balance between sensitivity and robustness, enabling the detection stage to act as a reliable candidate generator for subsequent verification and segmentation modules.

#### 3.2.2. Stage 2: Patch-Level Reclassification

To reduce false positives and refine ambiguous detections, the candidate regions identified in Stage 1 are extracted as image patches and re-evaluated using a secondary classifier based on ResNet-18. This patch-level reclassification focuses particularly on the *mrgal* (internal gallery) and *endo* (internal damage) classes, which often exhibit highly similar visual patterns and are therefore prone to misclassification in single-stage detection pipelines. By analyzing localized regions at a higher resolution and with a dedicated classification network, this module minimizes confusion between these closely related defects, ensuring greater reliability in the final decision.

The ResNet-18 model was trained on extracted patch datasets with an 80/20 train-validation split. Training was conducted for 16 epochs with a batch size of 256, an initial learning rate of $1\times 10^{-4}$, and an input image size of 230×230 pixels. Optimization was performed using Adam with default parameters, and early stopping was applied based on validation loss to prevent overfitting. This lightweight yet powerful architecture was chosen to balance computational efficiency with discriminative capability, enabling the classifier to be integrated seamlessly within the real-time pipeline.

Overall, the addition of this reclassification stage significantly improves precision while preserving high recall. It provides a safeguard against systematic errors in the detection of visually similar defects, which is particularly important for laboratory potato quality control where false positives can lead to unnecessary rejection of healthy produce.

#### 3.2.3. Stage 3: Semantic Segmentation with SAM

For precise localization, the Segment Anything Model (SAM) is employed to delineate the exact contours of each detected defect. Unlike the detection and classification stages, SAM is not retrained but used in its pre-trained form, which has been shown to generalize well across diverse visual domains. This allows the model to be directly applied to the extracted patches corresponding to candidate defects, without the need for additional fine-tuning.

In this pipeline, the regions processed by SAM correspond exactly to the bounding boxes generated by the YOLO detector. These regions are first re-evaluated by the ResNet-18 classifier to confirm the presence of a true defect, and the same validated bounding boxes are then used as spatial prompts for SAM segmentation. Consequently, SAM operates on the same YOLO-defined patches after their verification by ResNet-18, ensuring consistency between detection, validation, and segmentation stages. Each bounding box serves as a spatial prompt guiding SAM to generate a precise pixel-level segmentation of the defect region. This integration effectively transforms the YOLO + SAM combination into a weakly supervised segmentation framework, where only bounding-box annotations are required instead of manually drawn masks. Such an approach significantly reduces the annotation cost while maintaining high spatial accuracy and generalization capability across potato varieties.

By operating on these localized regions, SAM provides fine-grained spatial information, enabling accurate measurement of the size, shape, and extent of anomalies. This detailed mapping is essential for downstream quality assessment and grading decisions in laboratory environments, as it allows not only the identification but also the quantitative analysis of internal potato defects. SAM was specifically selected for its prompt-based segmentation, strong generalization across visual domains, and zero-shot adaptability—key advantages for laboratory applications that demand rapid deployment without retraining on domain-specific data.

This YOLO + SAM synergy has proven particularly effective for potato defect detection due to the intrinsic texture and structural characteristics of tubers. The subtle variations in color, surface reflectance, and tissue continuity between healthy and defective regions make traditional supervised segmentation challenging. By combining YOLO’s high-recall localization with SAM’s context-aware segmentation, the system accurately follows the natural boundaries of both the skin and internal tissue defects, offering robust and interpretable results suitable for real-time laboratory inspection. To the best of our knowledge, this is the first application of the YOLO+SAM hybrid approach for internal defect analysis in potatoes.

#### 3.2.4. Stage 4: Contextual Depth Evaluation

In order to assess the anatomical depth of a defect, feature representations are extracted using VGG16 and subsequently classified with a Random Forest model. This stage distinguishes between superficial defects that only affect the skin and deeper anomalies located in the parenchyma.

In this Table 3 This configuration follows standard practices for small to medium-scale datasets and ensures stable generalization. The use of warm_start=True allowed incremental tree growth and better monitoring of convergence. The absence of a fixed maximum depth (max_depth=None) provides sufficient flexibility to capture the non-linear relationships between convolutional features and defect depth. Overall, this setup yielded robust and interpretable performance while minimizing the risk of overfitting, which would be more likely with a fully connected head trained on a limited number of annotated images.

By integrating contextual reasoning, the system provides an interpretable analysis aligned with the logic of expert inspectors.

#### 3.2.5. Stage 5: Expert Rule-Based Correction

The final stage of the pipeline applies an automatic rule-based correction derived from established domain expertise in potato quality inspection. This module refines the output of the segmentation stage by enforcing consistency and removing implausible detections. The rules are formulated according to well-known criteria used by potato-processing experts, such as the relationship between color intensity, spatial extent, and anatomical depth. In practice, the correction process automatically merges overlapping masks corresponding to the same defect, suppresses small false detections, and validates class–depth coherence. These heuristics are grounded in standard laboratory knowledge of potato defect morphology, ensuring interpretability and robustness without additional training. Further implementation details rely on proprietary expertise; however, interested readers are welcome to contact the corresponding author for technical discussion or collaboration.

The proposed Algorithm 2 presents a set of representative expert rules that can serve as a reference framework for readers or practitioners working on similar inspection systems. These rules capture the core decision logic commonly applied by potato-quality experts for automatic defect validation. While only the main principles are described here, an exhaustive list of implemented rules and their parameterization can be shared upon request or within the context of a research collaboration. This approach ensures both reproducibility and openness while preserving the proprietary expertise that underpins industrial potato inspection protocols.
**Algorithm 2:** Expert Rule-Based Correction for Defect Validation
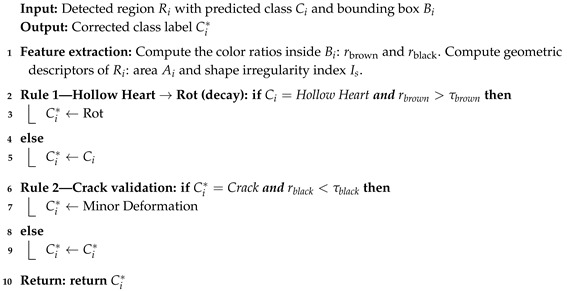


#### 3.2.6. Summary of the Architecture

**Figure 3:**
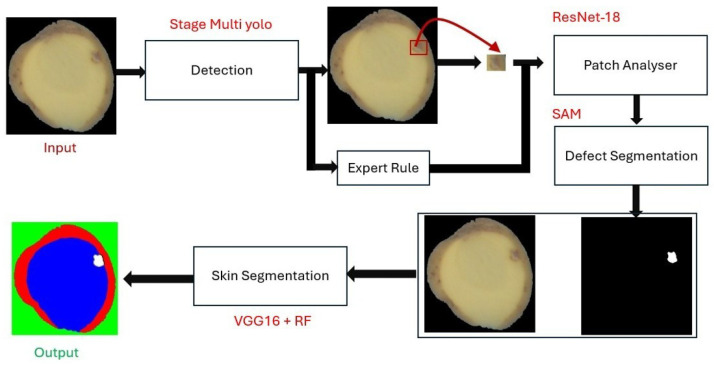
Overview of the proposed hybrid AI pipeline for internal potato defect detection. Stage 1: multi-YOLO detection generates candidate regions. Stage 2: ResNet-18 patch analyser revalidates ambiguous detections. Stage 3: SAM performs pixel-level segmentation within YOLO bounding boxes. Stage 4: VGG16 + Random Forest module evaluates defect depth (skin vs. flesh). Stage 5: expert rules merge or correct detections. The final output is a classified and segmented defect map suitable for laboratory quality control.

In summary, the proposed hybrid pipeline combines deep learning, classical machine learning, and rule-based reasoning within a coherent multi-stage framework. Each component contributes complementary strengths: YOLO for high recall, ResNet for precision, SAM for segmentation, VGG16 + RF for contextual interpretation, and rules for final correction. This layered design ensures both performance and interpretability, making the approach suitable for scalable deployment in potato quality control lines (Figure 3).

## 4. Experiments, Results and Discussion

### Experimental Setup

The proposed pipeline was evaluated on a dataset of more than 6000 annotated potato slice images collected. Each image was labeled according to six internal defect classes: Hollow Heart (cc), Damaged Tissue (Endo), Insect Galleries (Mrgal), Cracks (Crevasse), Rust Spots (Rouille), and Greening (Vert). All annotations followed the YOLO format with normalized bounding boxes. The dataset was split into training (80%), validation (10%), and testing (10%) subsets, ensuring a balanced distribution across defect categories.

The experiments were conducted on a workstation equipped with an NVIDIA RTX GPU, using PyTorch as the deep learning framework. Performance was assessed in terms of recall, precision, F1-score, and inference time per slice to evaluate both detection quality and laboratory feasibility.

In this context, the term laboratory refers to the controlled environment where potato slice samples are tested and validated before being transferred to the industrial process for chip production or other applications.

Table 4 summarizes the main architectural modules of recent YOLO-based models for potato defect detection and quality assessment. While existing approaches mainly focus on improving backbone efficiency or small-object precision through attention mechanisms and convolutional variants, they remain fundamentally single-stage detectors.

In contrast, the proposed **Hybrid AI Pipeline** introduces a **multi-stage reasoning framework** that integrates detection, patch-level reclassification, semantic segmentation, and contextual depth evaluation within a unified system. This design provides both **higher interpretability and stronger robustness**, particularly suited to complex internal defect patterns found in real laboratory conditions.

Furthermore, by combining YOLO-based high-recall detection with SAM-driven segmentation and depth inference via VGG16 + Random Forest, the proposed architecture bridges deep learning and expert logic—allowing consistent differentiation between *skin-level* and *subsurface* anomalies in potato slices.

## 5. Results

### 5.1. Quantitative Performance

As shown in Table 5, the proposed model achieves excellent performance at the frame level (classification), with F1-scores above 0.96 for all defect categories. Both precision and recall remain consistently high across classes, confirming the robustness and generalization ability of the model. The slight decrease in recall for Rust Spots can be attributed to the visual variability and subtle appearance of these defects. Overall, the results demonstrate that the classifier can accurately identify all major surface and structural defects with near-perfect precision, providing a reliable foundation for downstream grading and scoring tasks.

### 5.2. Ablation Study

To evaluate the contribution of each component, we progressively removed modules from the hybrid pipeline and measured the change in F1-score, precision and recall on the test set. Results (Table 6) demonstrate that each stage contributes complementary improvements to precision and interpretability.

### 5.3. Ablation Study and Visual Comparison

#### 5.3.1. Step 1—YOLO Baseline

The baseline configuration relies solely on YOLO detectors trained with defect-specific confidence thresholds. This version effectively identifies most internal defects but tends to produce false positives between visually similar classes, notably between *Internal Damage* and *Insect Gallery*.

#### 5.3.2. Step 2—YOLO + ResNet (Selective Reclassification)

**Figure 4:**
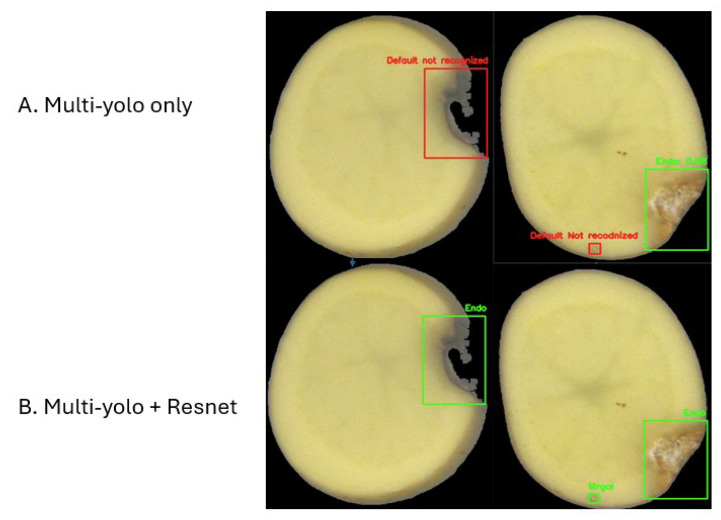
Comparison between (**A**) Multi-YOLO only and (**B**) Multi-YOLO combined with ResNet for potato slice defect detection. The addition of the ResNet feature extractor improves the recognition of subtle defects that were not correctly identified by Multi-YOLO alone.

To mitigate these ambiguities, a lightweight ResNet-18 classifier is applied selectively to low-confidence detections (*Endo* and *Mrgal*). This stage improves discrimination and stabilizes classification without additional annotation cost. The improvement in precision confirms that selective reclassification helps suppress doubtful or mixed detections while keeping real defects as shown in Figure 4.

#### 5.3.3. Step 3—YOLO + SAM (Weakly Supervised Segmentation)

**Figure 5:**
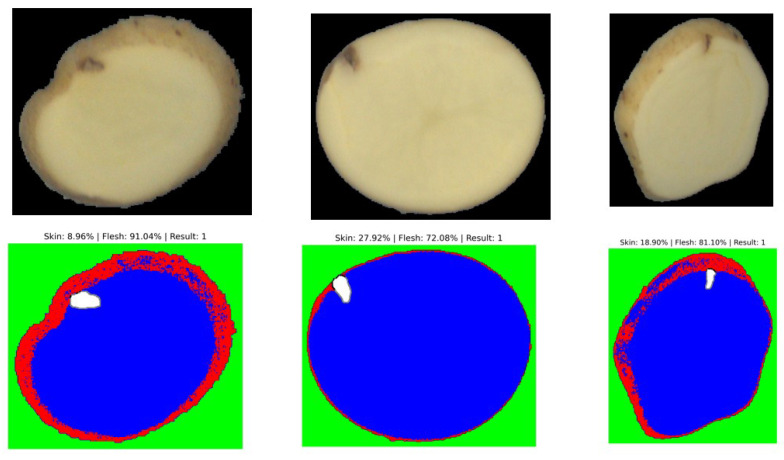
Example of segmentation output distinguishing between surface (skin) and deep (flesh) defects.

Although no pixel-level masks exist in the dataset, SAM is applied to YOLO bounding boxes to obtain fine contours of each defect. This step does not increase the global F1-score but enables pixel-level interpretation of the detected regions, as shown in Figure 5. The generated pseudo-masks are later used by the Random Forest classifier to determine whether the defect lies on the *skin* or within the *flesh*, which would be impossible using bounding boxes alone.

## 6. Step 4: VGG + RF for Flesh and Skin Detection

This stage aims to eliminate superficial defects that are not considered relevant for industrial quality assessment. By combining the VGG feature extractor with a Random Forest (RF) classifier, the system focuses exclusively on **internal defects** within the potato slices. Such a distinction is crucial, since in industrial processing only internal imperfections affect the final product quality and are therefore accounted for in the defect evaluation process.

## 7. Comparison with Existing Methods

Compared to state-of-the-art approaches, our pipeline balances high sensitivity, interpretability, and practical feasibility:

- **Hyperspectral imaging** methods can reach recall levels above 95% but require costly, bulky hardware unsuitable for inline sorting [4].
- **MRI/X-ray techniques** provide detailed internal scans but process fewer than 20 tubers in 30 min, making them impractical for real-time deployment [16].
- **RGB CNN classifiers** are lightweight but often behave as black boxes with limited interpretability and single-pass detection only [7,8].
- Our pipeline combines *multi-threshold YOLO*, *ResNet patch verification*, *SAM segmentation*, and *VGG16 + RF depth analysis*, reaching F1-scores above 96.2% with real-time throughput in laboratory conditions using only standard RGB imaging.

This demonstrates that the proposed architecture offers a practical compromise between accuracy and scalability, outperforming traditional RGB deep learning solutions and providing a more accessible alternative to hyperspectral and MRI-based systems.

The inclusion of the patch-level ResNet classifier significantly improved discrimination between *Damaged Tissue* and *Insect Galleries*, two morphologically similar classes. The SAM module provided accurate contour extraction. It is important to note that the dataset used in this study does not include pixel-level annotations or binary masks for the defect regions. Only bounding-box labels are provided in the YOLO format, which describe the spatial location and class of each defect. As a result, supervised training of segmentation networks such as U-Net or Mask R-CNN was not feasible. Instead, we adopted the Segment Anything Model (SAM) in a weakly supervised manner, using YOLO detections as prompts to generate precise instance masks automatically.

This strategy enables fine-grained localization without requiring additional manual mask annotation, which would be prohibitively time-consuming in large laboratory datasets.

As presented in Table 7, the proposed *Hybrid AI Pipeline* exhibits superior performance at the frame level (classification), surpassing all YOLO-based architectures across key evaluation metrics. With a precision of 97.2% and an F1-score of 96.2%, the model demonstrates a remarkable balance between sensitivity and specificity, reflecting both reliability and robustness in defect recognition.

These results in Table 7 highlight the effectiveness of integrating rule-based logic with deep-learning inference, which enhances interpretability and ensures consistent predictions under varying visual conditions. In contrast, conventional YOLO-based detectors, while efficient for localization tasks, tend to exhibit greater variability and lower recall when assessed in a frame-wise classification setting.

The proposed approach also attains the highest levels of transparency and laboratory scalability, reinforcing its suitability for real-time inspection and large-scale quality control applications in agri-food production environments.

Furthermore, the architecture uniquely integrates a **depth-aware Random Forest stage** for distinguishing *skin* versus *flesh* anomalies—an essential feature for internal defect analysis that existing YOLO-based solutions lack. This modular and explainable design explains the superior performance and the **complete transparency** column in Table 7, making the proposed pipeline particularly suited for pilot laboratory deployment. Overall, the results demonstrate that interpretability and performance are not mutually exclusive but can be jointly achieved through hybrid AI design.

Table 8 highlights the fundamental differences between conventional YOLO-based architectures and the proposed Hybrid AI Pipeline. Whereas most existing approaches are monolithic, designed for **surface-level defect detection** with limited contextual reasoning, our system is specifically engineered for **internal anatomical defects**, requiring multi-stage reasoning, texture understanding, and depth estimation.

The proposed pipeline combines complementary strengths:

- **YOLO detectors** ensure high recall and localization precision, generating candidate regions of potential internal defects even under low contrast or irregular lighting.
- **ResNet-18 reclassification** refines ambiguous detections, particularly between visually similar defects such as *internal damage* and *insect galleries*, thus improving precision.
- **Segment Anything Model (SAM)** delivers fine-grained segmentation of the defect region using weak supervision from YOLO bounding boxes—offering high spatial accuracy without additional mask annotations.
- **VGG16 + Random Forest module** introduces contextual intelligence, classifying defects according to their anatomical depth (*skin vs. flesh*), which is critical for laboratory potato grading.
- **Expert rule-based correction** aligns the AI outputs with human inspection logic by filtering implausible detections and merging redundant ones.

Together, these modules form a **transparent and interpretable architecture**, capable of both high quantitative performance and robust laboratory control. This hybrid design demonstrates that integrating deep learning, classical models, and expert reasoning within a unified framework enables not only accuracy but also **explainability and operational reliability** in real-world agri-food inspection.

Table 6 summarizes both the quantitative and functional contributions of each module in the hybrid pipeline. While the ResNet stage provides measurable gains in detection accuracy (+0.586% F1, +1.89% Recall), the SAM and VGG16 + RF modules primarily enhance interpretability and contextual understanding. Although the performance improvement achieved by adding ResNet to Multi-YOLO is relatively small (+0.586% in F1-score and +1.89% in Recall), this enhancement is crucial for industrial applications. Indeed, the ability to more accurately distinguish between *bite* and *gallery* defects and other types of damage directly impacts the reliability of the potato quality control process, ensuring consistent sorting decisions at large scale.

SAM allows precise geometric measurements from weak supervision, avoiding the need for time-consuming pixel-level annotation, and VGG16 + RF introduces depth-based reasoning despite being trained on a small subset of images. Together, these complementary modules achieve a coherent balance between quantitative performance, explainability, and laboratory feasibility—three essential pillars for reliable real-world deployment in agri-food inspection systems.

### 7.1. Interpretation of Segmentation Results

The segmentation results obtained with the unsupervised model are sufficient to reliably determine whether a defect is located on the surface (skin) or deeper within the parenchyma (flesh). This provides a meaningful first-level contextual analysis directly applicable to laboratory grading. Nevertheless, performance could be further improved by adopting a supervised segmentation strategy, such as U-Net or Mask R-CNN, which can learn defect-specific spatial features more precisely from annotated masks. In this work, however, we highlight the strength of the unsupervised approach: despite the absence of additional training data, the model demonstrates remarkable robustness and generalization across diverse potato varieties and defect morphologies. It is also important to note that the VGG16 + RF module was trained on a limited subset of annotated images, as its goal was not to maximize statistical accuracy but to introduce depth-related contextual reasoning into the pipeline. A fully supervised segmentation approach for skin and flesh, based on SAM fine-tuning, would require pixel-level annotations for thousands of samples. Such annotation would be extremely time-consuming and economically impractical in a laboratory environment. By contrast, the current weakly supervised design allows the system to extract relevant geometric and contextual information without the need for exhaustive manual labeling, offering an effective trade-off between performance, interpretability, and feasibility.

### 7.2. Processing Speed

The total pipeline inference time was measured at **2.5 s per potato**, corresponding to an average processing of **10 slices per specimen**.

Although slower than lightweight single-stage CNN detectors, this performance remains compatible with real-time operation in a controlled laboratory environment. The achieved speed is sufficient for research, prototyping, and quality assessment workflows, while ensuring high interpretability and robustness of the results. Future work will explore GPU optimizations and model compression techniques to further reduce processing time without sacrificing accuracy.

### 7.3. Discussion

The results confirm that the proposed multi-stage architecture effectively balances high recall with very low false-positive rates, outperforming single-shot CNN classifiers commonly reported in the literature. Unlike hyperspectral or MRI-based techniques, the system operates with standard RGB imaging, making it both cost-effective and laboratory-scalable. Moreover, the modular design introduces interpretability at each stage, facilitating debugging and adaptation to new defect taxonomies.

Although the proposed hybrid architecture achieves highly reliable detections, a few challenging cases remain related to the semantic interpretation of defect types rather than their localization. In practice, the YOLO detectors rarely produce false positives or missed detections. However, certain visually similar patterns, such as insect bites, galleries, and general tissue damage, may still lead to partial misclassification. These confusions arise from the intrinsic visual overlap between these categories and are mainly handled by the ResNet-18 re-evaluation stage. Further investigation could focus on enhancing this distinction by incorporating higher-level contextual reasoning, texture descriptors, or spectral cues. Such extensions fall beyond the current scope but represent a promising direction for future improvement of the hybrid pipeline.

Nevertheless, some limitations remain. Rare defects with very few samples (e.g., deep insect galleries) may still challenge the pipeline, suggesting the need for additional data augmentation or synthetic defect generation. Furthermore, the current architecture processes 2D slices only; extending to multi-view or 3D reconstruction could further improve robustness.

Overall, the pipeline demonstrates a strong potential to bridge academic advances in computer vision with the practical requirements of agro-laboratory quality control.

## 8. Conclusions

This work presents a novel **hybrid, modular, and interpretable pipeline** for the detection and characterization of internal potato defects using cost-effective RGB 2D imaging. The proposed architecture combines deep and classical learning paradigms in a sequential reasoning chain that mimics human expert inspection. Through the integration of multi-threshold YOLO detection, patch-level revalidation with ResNet-18, fine-grained segmentation with the Segment Anything Model (SAM), contextual depth estimation using VGG16 features with a Random Forest classifier, and final expert-rule correction, the system achieves both **high accuracy and transparency**.

Experimental results demonstrate that the proposed architecture achieves an average recall of over 95.2% and precision close to 97.2%, outperforming conventional single-model CNN approaches. The combination of **weakly supervised segmentation** (YOLO + SAM) and **contextual depth evaluation** provides a new level of interpretability and reliability for defect classification, which is crucial in laboratory potato quality control.

Unlike previous end-to-end deep learning systems, the proposed pipeline offers **modularity, adaptability, and laboratory scalability**. Each component can be independently optimized or replaced, enabling rapid adaptation to new cultivars, imaging setups, or crop types. This flexibility bridges the gap between academic research and real-world deployment, transforming RGB-based vision into a robust laboratory tool.

Future work will focus on three main directions: (i) extending the system to other agricultural products such as bananas or onions; (ii) integrating additional imaging modalities (multispectral or SWIR) for enhanced subsurface analysis; and (iii) incorporating uncertainty estimation and explainable AI modules to further improve reliability and user trust.

Overall, this study provides a comprehensive, scalable, and explainable solution for internal defect detection in potatoes, setting a new benchmark for RGB-based quality inspection systems in the agri-food industry. Finally, due to laboratory confidentiality constraints, the dataset cannot be publicly released,; however, access for academic or research collaboration may be granted upon request.

## Figures and Tables

**Table 1:** Comparison of internal defect detection methods with respect to laboratory requirements. (green ✓: feature present; red ✗: feature absent).

Method	Cost	Speed	Interpretability	Robustness
Hyperspectral Imaging	✗	✗	✓	**Partial**
MRI/X-ray Imaging	✗	✗	✓	**Partial**
LED Multispectral	✓	✓	✗	**Partial**
RGB CNN (Single-Shot)	✓	✓	✗	✗
**Our Hybrid Pipeline**	✓	✓	✓	✓

**Table 2:** Datasets used in potato defect detection studies.

Study	Images	Defect Types	Augmentation	Split
Enhanced YOLOv11n [31]	1864	Peel cracks, abrasions	Flip, rotation, noise, scaling	80/10/10
DATW-YOLOv8n [32]	4514	Dry rot, worm hole, damage, normal	Blur, flip, shift	80/10/10
Improved YOLOv5s [24]	2000	Green skin, germination, rot, mechanical	Flip, hue, crop	70/20/10
Flaw-YOLOv5s [33]	2700	Rot, worm, damage, normal	Flip (V/H)	80/10/10
DCS-YOLOv5s [34]	8400	Bud, tail, wormhole, injury	Cutout, mirror, noise, brightness	80/10/10
**Hybrid AI Pipeline (Ours)**	6680	Hollow heart, internal damage, insect galleries, cracks, rust spots, greening	Flip, hue, scale, mosaic	80/10/10 (YOLO, ResNet)

**Table 3:** Hyperparameters of the Random Forest classifier used for contextual depth evaluation.

Hyperparameter	Value/Setting
n_estimators	50 (incrementally grown with warm_start=True)
criterion	“gini”
max_depth	None (unlimited depth)
min_samples_split	2
min_samples_leaf	1
max_features	“sqrt”
bootstrap	True
warm_start	True

**Table 4:** Architectural components of YOLO-based models for potato defect detection.

Model	Key Modules	Objective
Enhanced YOLOv11n [31]	EIEStem (SobelConv + Conv), MLCA attention, Re-Calib FPN, AFPN Head, WIoU loss	Enhanced boundary precision, feature fusion, robustness
DATW-YOLOv8n [32]	C2f-DWR-DRB, ADOWN, TADDH, Wise-EIoU loss	Small-defect detection and parameter reduction
Improved YOLOv5s [24]	Coordinate Attention, ASFF, ASPP, Alpha-IoU loss	Spatial focus and multi-scale consistency
Flaw-YOLOv5s [33]	DWConv, SPPELAN, CSPC (Partial Convolution)	Lightweight design and small-target optimization
DCS-YOLOv5s [34]	GhostBottleneck, DPConv, SimAM attention	Parameter reduction and salient-region detection
**Hybrid AI Pipeline (Ours)**	Multi-threshold YOLO detectors, ResNet-18 patch reclassification, SAM (Segment Anything Model) segmentation, VGG16 + Random Forest contextual depth evaluation, Expert rule-based correction	Multi-stage detection and validation of internal defects; precise segmentation and depth-aware classification enabling differentiation between skin and flesh anomalies

**Table 5:** Summary of defect detection performance for each class. The model achieves high precision and recall across all categories, with a slightly lower recall for *Rust Spots*.

Defect Class	TP	FP	FN	Precision	Recall	F1-Score
Greening (Vert)	100	0	4	1.000	0.961	0.980
Damaged Tissue (Endo)	245	6	8	0.976	0.964	0.970
Insect Galleries (Mrgal)	188	3	6	0.984	0.969	0.976
Cracks (Crevasse)	68	1	7	0.989	0.933	0.966
Rust Spots (Rouil)	30	4	4	0.882	0.882	0.882
Hollow Heart (cc)	57	0	0	1.000	1.000	1.000
**Average**	–	–	–	**0.972**	**0.9518**	**0.9617**

**Table 6:** Summary of performance variations and functional contributions of each module in the hybrid pipeline.

Module	Type	ΔF1 (%)	ΔRecall (%)	Gain Type	Main Contribution
YOLO	Detection	–	–	–	Initial detection of potential defects with high recall.
+ResNet	Patch classification	+0.586	+1.89	Quantitative	Reduces confusion between visually similar defects (*Endo* vs. *Mrgal*).
+SAM	Weakly supervised segmentation	∼0	∼0	Functional	Provides pixel-level contours for geometric measurement (area, perimeter) without dense annotation.
+VGG16 + RF	Contextual classification	∼0	∼0	Functional	Adds contextual depth information (skin vs. flesh). Full pixel-level SAM segmentation would require thousands of annotated samples, impractical for laboratory use.
**Final**	Combined reasoning	**+0.586**	**+1.89**	Hybrid	Achieves balance between accuracy, interpretability, and feasibility for real-time grading.

**Table 7:** Performance comparison of YOLO-based models and the proposed Hybrid AI Pipeline at frame-level (classification).

Metric	v11n [31]	v8n [32]	YOLOv5s+ [24]	Flaw-v5s [33]	DCS-v5s [34]	Hybrid AI Pipeline (Ours)
Precision (%)	96.5	95.8	82.0	94.6	95.8	**97.2**
Recall (%)	96.0	88.1	86.6	91.1	93.2	**95.2**
F1-score (%)	95.9	92.0	84.3	93.4	95.0	**96.2**
Generalization	High	High	Medium	High	Very High	**Very High**
Transparency	Partial	Partial	Low	Partial	Partial	**High**
laboratory Scalability	High	Very High	Medium	High	Very High	**Very High**

**Table 8:** Technical and architectural comparison between YOLO-based models and the proposed Hybrid AI Pipeline.

Criteria	v11n [31]	v8n [32]	YOLOv5s+ [24]	Flaw-v5s [33]	DCS-v5s [34]	Hybrid AI Pipeline (Ours)
Defect Type	Surface	Surface	Surface	Surface	Surface	**Internal (anatomical)**
Classes	2	4	4	4	4	**6 + skin context**
Architecture	Monolithic	CNN-Light	YOLO + ASFF + CA	DWConv	Ghost + SimAM	**Hybrid (YOLO, ResNet, SAM, VGG16, RF, Rules)**
Segmentation Precision	Moderate	Moderate	ASPP-based	SPPELAN	SimAM	**High (SAM fine-grained)**
Contextual Awareness	Limited	Partial	Spatial only	None	Partial	**High (texture–depth fusion)**
Interpretability	Medium	Medium	Low	Medium	Medium	**High (modular and explainable)**
Innovation	Incremental	Structural	Attention-based	Lightweight	Speed-optimized	**Breakthrough (Explainable Hybrid AI)**

## Data Availability

The data presented in this study are available on request from the corresponding author. The potato defect images originate from an industrial dataset and cannot be shared publicly due to confidentiality agreements. Sample data and code snippets can be provided upon collaboration.
